# Observation of the influence of dipolar and spin frustration effects on the magnetocaloric properties of a trigonal prismatic {Gd_7_} molecular nanomagnet†
†Electronic supplementary information (ESI) available: Synthesis, crystallographic, magnetic measurements and numerical simulations details and figures. CCDC 1458052. For ESI and crystallographic data in CIF or other electronic format see DOI: 10.1039/c6sc01415a


**DOI:** 10.1039/c6sc01415a

**Published:** 2016-04-25

**Authors:** Eufemio Moreno Pineda, Giulia Lorusso, Karzan H. Zangana, Elias Palacios, Jürgen Schnack, Marco Evangelisti, Richard E. P. Winpenny, Eric J. L. McInnes

**Affiliations:** a School of Chemistry and Photon Science Institute , The University of Manchester , Oxford Road , Manchester M13 9PL , UK . Email: Eric.Mcinnes@manchester.ac.uk ; Email: richard.winpenny@manchester.ac.uk; b Instituto de Ciencia de Materiales de Aragón (ICMA) , CSIC – Universidad de Zaragoza , Departamento de Física de la Materia Condensada , 50009 Zaragoza , Spain; c Department of Chemistry , College of Education , Salahaddin University-Erbil , Kurdistan Region-Iraq; d Faculty of Physics , University of Bielefeld , Universitätsstr. 25 , D-33615 Bielefeld , Germany

## Abstract

We report the synthesis and structure of a molecular {Gd_7_} cage of the formula (^i^Pr_2_NH_2_)_6_[Gd_7_(μ_3_-OH)_3_(CO_3_)_6_(O_2_C^*t*^Bu)_12_] which has crystallographic *C*_3h_ symmetry. Low temperature specific heat and adiabatic demagnetization experiments (the latter achieving temperatures below 100 mK), lead to the observation of the effects of both intramolecular dipolar interactions and geometric spin frustration. The dipolar interaction leads to a massive rearrangement of energy levels such that specific heat and entropy below 2 K are strongly modified while magnetic susceptibility and magnetization above 2 K are not affected. The consequences of these phenomena for low temperature magnetocaloric applications are discussed.

## Abstract 2

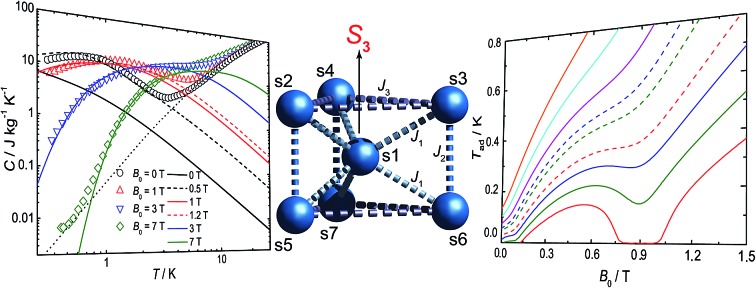
Adiabatic demagnetisation experiments on a *C*_3h_ symmetric molecular {Gd7} cluster reveal effects of intramolecular dipolar interactions and geometric spin frustration.

## Introduction

A wide variety of applications have been envisioned exploiting the magnetic properties of mono- or poly-metallic lanthanide complexes.^1^ The anisotropic lanthanides have been proposed as building blocks in quantum computers,^2^ data storage^3^ and spintronic devices.^4^ Conversely, Gd(iii) systems, where the lanthanide ion is isotropic and has a large spin (*s* = 7/2), can have large magnetocaloric effects (MCE),^5^ and hence could be good candidates for very low temperature magnetic cooling.^6^

Almost all MCE studies of molecular clusters have been indirect, where magnetization or heat capacity data are analysed to derive magnetic entropy changes for a given change in applied magnetic field. Some of us recently reported direct magnetocaloric experiments on a {Gd_7_} molecule where we achieved cooling to temperatures as low as ∼200 mK in adiabatic demagnetization experiments.^7^ Moreover, we found that the temperature evolution of the sample on demagnetisation under quasi-adiabatic conditions (following paths of constant entropy or isentropes) was not linear as for a simple paramagnet, but showed specific regions of enhanced cooling, and even heating, on demagnetisation. That {Gd_7_} molecule has a centered-hexagonal array of metal ions. With antiferromagnetic exchange coupling, this triangle-based structure suffers geometric spin frustration. The latter is an important phenomenon in extended lattices, and is associated with having many degenerate ground state configurations, leading to exotic magnetic behaviours such as spin glasses and spin ice.^8^ It can also lead to enhanced MCE because of the increased density of states at critical fields.^9^ We showed that the observed isentrope structure in {Gd_7_} was a direct signature of spin frustration.^7^

Here we report a new {Gd_7_} cage with a different geometrically frustrated structure and investigate the consequences on its MCE *via* adiabatic demagnetization experiments. Moreover, we observe the effects of internal dipolar interactions that are competitive with the exchange couplings, with further consequences for MCE applications.

## Results and discussion

**Fig. 1:**
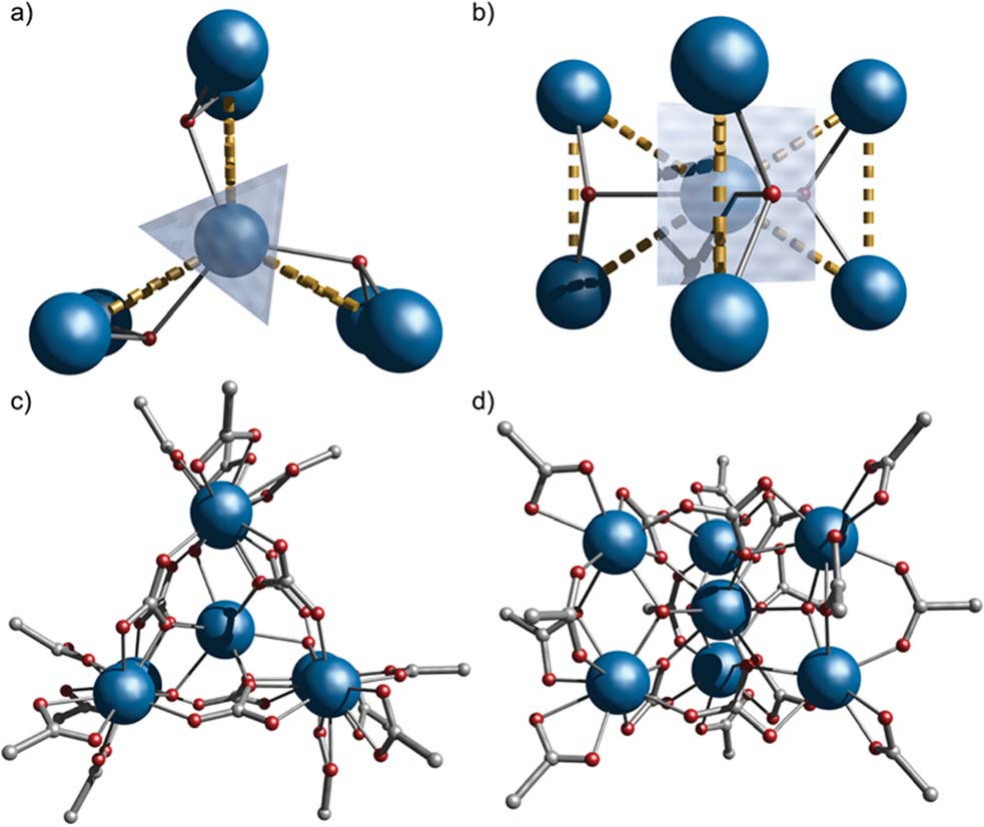
Crystal structure of **1**, viewed down (a and c) and perpendicular to (b and d) the *S*_3_ axis (Gd, blue; O, red; C, grey; H and Me omitted for clarity). Dashed lines highlight three {Gd_3_} triangles sharing a vertex.

The compound is obtained from reaction of [Gd_2_(O_2_C^*t*^Bu)_6_(HO_2_C^*t*^Bu)_6_]^10^ with ^i^Pr_2_NH in MeCN (see ESI for more details†). Single crystal X-ray studies showed formation of (^i^Pr_2_NH_2_)_6_[Gd_7_(μ_3_-OH)_3_(CO_3_)_6_(O_2_C^*t*^Bu)_12_]§
§Crystal data for **1** [C_106_H_210_Gd_7_O_45_N_8_]: *M*_r_ = 3417.56, hexagonal, *T* = 150.0(2) K, *a* = 16.9152(2), *c* = 31.3646(6) Å, *V* = 7771.9(3) Å^3^, *Z* = 2, *ρ* = 1.460 g cm^–3^, total data = 43 084, independent reflections 5409 (*R*_int_ = 0.0339), *μ* = 3.013 mm^–1^, 305 parameters, *R*_1_ = 0.0310 for *I* ≥ 2*σ*(*I*) and w*R*_2_ = 0.0637, deposit number CCDC 1458052. The data was collected on an Agilent SuperNova CCD diffractometer with MoK_α_ radiation (*λ* = 0.71073 Å), solved using SUPERFLIP,17*a* and refined on *F*^2^ using SHELX-14 (ref. 17*b*) in Olex2.17*c*
**1** (Fig. 1).

**1** crystallizes in the *P*6[combining macron]2*c* space group with the anionic {Gd_7_} cage lying on the 6[combining macron] (*S*_3_) axis, giving one sixth of the molecule in the asymmetric unit, and hence crystallographic *C*_3h_ symmetry. The metallic core consists of six Gd(iii) ions forming a trigonal prism [Gd(2) and symmetry equivalents]. The prism encapsulates a central Gd(iii) ion [Gd(1), Fig. 1]. Six CO_3_^2–^ groups bridge the trigonal edges of the prism, also binding to the central Gd (3.211 binding in Harris notation);^11^ these carbonates must arise from CO_2_ fixation.^12^ There are three μ_3_-OH, one bridging each edge between the triangular faces of the trigonal prism, also binding to the central Gd(iii) ion. This highlights an alternative description of the cage as three {Gd_3_(μ_3_-OH)} triangles sharing a single vertex [Gd(1), Fig. 1b]: the triangles are isosceles with Gd(1)···Gd(2,2′) and Gd(2)···Gd(2′) distances of 3.9548(1) and 3.9388(9) Å, respectively. Twelve pivalates complete the cage: two pivalates bridge each Gd(2)···Gd(2′) with 2.11 and 2.21 coordination modes (the latter is disordered, lying on the mirror plane), and each Gd(2) vertex is capped by a 1.11 pivalate. Gd(1) is nine-coordinate with tricapped trigonal-prismatic geometry (continuous shape measure, CShM of 0.910, Table S1†).^13^ Gd(2) is eight-coordinate, but no clear unique description is favoured by CShM (Table S1†). Six di-isopropyl ammonium cations provide charge-balance.

**Fig. 2:**
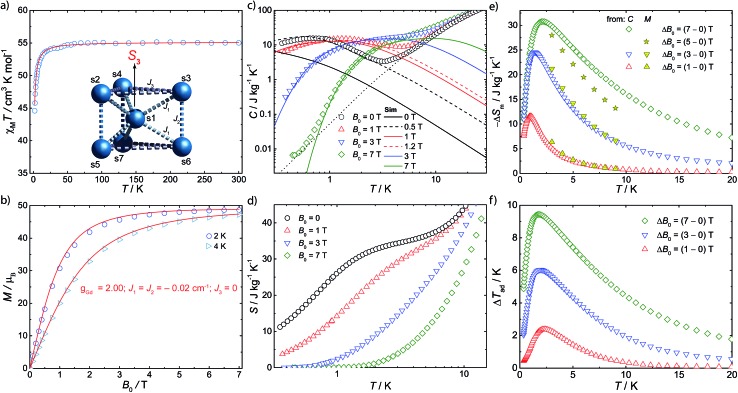
Magnetic properties of **1**. (a) Molar magnetic susceptibility (*χ*_M_), in the form of *χ*_M_*T*(*T*), measured in an applied magnetic field of 0.1 T, and fit (solid line) to spin Hamiltonian (1) appropriate for the spin system in the insert. (b) Magnetization (*M*) as a function of applied field (*B*_0_) and temperature (*T* = 2, 4 K), and fits (solid lines) from spin Hamiltonian (1). (c) Specific heat (*C*) as a function of temperature at *B*_0_ = 0 (black symbols), 1 T (red), 3 T (blue) and 7 T (green), and lattice contribution (dotted line). Solid lines show the results that follow from Hamiltonian (1), dashed lines those including an effective internal field, see text. (d) Entropy, as obtained from *C*(*T*) data. (e) Magnetic entropy change obtained from *C*(*T*,*B*_0_) and *M*(*T*,*B*_0_). (f) Adiabatic temperature change obtained from *C*(*T*,*B*_0_).

Magnetic susceptibility (*χ*_M_) studies (applied magnetic field, *B*_0_ = 0.1 T) were carried out on a polycrystalline sample of **1** in the temperature (*T*) range 2–300 K (Fig. 2a). At room-temperature the product *χ*_M_*T* is 55.2 emu K mol^–1^, consistent with seven Gd(iii), ^8^S_7/2_ ions (*χ*_M_*T* = 55.1 emu K mol^–1^ for seven non-interacting *s* = 7/2 with *g* = 2.00). Upon cooling, *χ*_M_*T*(*T*) remains constant down to *ca.* 10 K where it drops sharply, to 43.5 emu K mol^–1^ at 2 K. The magnetization (*M*) data for **1** at low temperature show rapid increases of *M* with applied magnetic field, reaching a saturation value of 48.7 *μ*_B_ at 7 T and 2 K (Fig. 2b and S1†), consistent with complete polarization of the spin system (maximum possible *M* is 49.0 *μ*_B_ for seven *s* = 7/2 with *g* = 2.00).

Specific heat (*C*) measurements under different applied magnetic fields were also carried out for **1** (Fig. 2c). Above *ca.* 5 K, *C* is dominated by lattice phonon modes of the crystal, which can be described by the Debye model (dotted line) and simplify to *C*/*T*^3^ = 0.05 J kg^–1^ K^–1^ at the lowest temperatures. The strong field dependence of *C* at low temperature, and the ability to fully magnetise the system, suggests **1** as a good candidate for a cryogenic magnetic refrigerant. Therefore, we have evaluated the entropy (Fig. 2d) and MCE (Fig. 2e and f) of **1** indirectly from *C*(*B*_0_,*T*) and *M*(*B*_0_,*T*), using known procedures.5*c* We obtain a maximum magnetic entropy change of –Δ*S*_m_ = 30.8 J kg^–1^ K^–1^ at 2.1 K for a field change Δ*B*_0_ = (7–0) T. Hence, under these experimental conditions, we are accessing a large proportion (83%) of the full magnetic entropy content, *viz. S*_m_ = *nR* ln(2*s* + 1) = 36.9 J kg^–1^ K^–1^ (*n* = 7, *s* = 7/2, *R* is the gas constant). The latter is reached in zero field for *T* above *ca.* 3 K (Fig. 2d), while nil magnetic entropy (*i.e.* magnetic saturation) is reached at 7 T below *ca.* 2 K. We can access this high fraction of the magnetic entropy because of the small antiferromagnetic exchange between Gd(iii) ions, coupled with spin frustration, giving a high density of low-lying states.

The magnetic data have been modelled using a Heisenberg spin Hamiltonian (1) consistent with the *C*_3h_ symmetry (Fig. 2a, insert):1
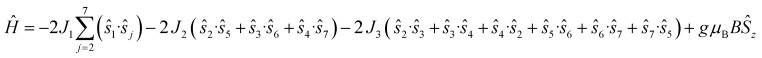


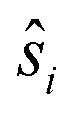
 denote the individual spin operators (*i* = 1 is the central Gd) and 
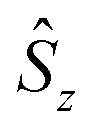
 denotes the *z*-component of the total spin operator. Since the Heisenberg Hamiltonian possesses several symmetries, a complete matrix diagonalization of the system is possible despite the huge 8^7^ matrix dimension.^14^*J*_1_ is the interaction between the central Gd ion (*S*_1_) and all others; *J*_2_ is the interaction on the edges between triangular faces of the trigonal prism; *J*_3_ is the interaction on the edges of the triangular faces. The best agreement between experimental data and calculations for *χ*_M_*T*(*T*) and *M*(*B*_0_) is achieved with *J*_1_ = *J*_2_ = –0.02 cm^–1^ and *J*_3_ = 0 cm^–1^, assuming *g* = 2.00 (Fig. 2a and b). Note that these parameters, with *J*_3_ = 0, would support the description of **1** as three triangles sharing a vertex as depicted in Fig. 1(a and b).

This topology results in geometric spin frustration because not all the antiferromagnetic interactions can be satisfied,^15^ and results in very high degeneracies of states. For example, for these parameters in this Heisenberg model, the three lowest energy eigenstates possess total spins of *S* = 13/2, 11/2 and 15/2, each with their 2*S* + 1 degeneracy, and lie within 0.005 cm^–1^ of each other. However, using this model to calculate the zero-field specific heat as a function of temperature gives a peak in *C*(*T*) at *ca. T* = 0.1 K which is not observed experimentally (Fig. 2c). This suggests that our model, at least below 2 K, is inadequate.

The isotropic exchange interactions found using eqn (1) are very weak, in fact significantly smaller than in other Gd(iii) cages we have studied (*e.g. J* ≈ –0.06 cm^–1^ in our planar {Gd_7_} that also contains μ_3_-OH bridges).^7^ Hence we calculated the intra-molecular dipolar interactions to test the validity of neglecting them. In fact for the shortest Gd···Gd distances (on the edges between triangular faces of the trigonal prism) the dipolar interaction tensor has elements that range in magnitude from 0.014 to 0.033 cm^–1^, and are thus of the same order as the isotropic *J* values found using (1). Hence they must be included in our model. However, quantum mechanical calculations with anisotropic Hamiltonians for spin systems of this size need millions of CPU hours on supercomputers,^16^ hence we have performed simulations with a fictitious cluster having the same magnetic skeleton as **1**, but replacing the *s* = 7/2 spins with spins *s* = 3/2.

Such simulations (Fig. S2 and S3, ESI†) show that the dipolar interactions influence the magnetic properties only mildly for *T* > 2 K, *i.e.* they do not significantly influence our modelling of the experimental *χ*_M_*T*(*T*) and *M*(*B*_0_) curves, and hence we would obtain the same best values for the isotropic *J* as above. However, inclusion of dipolar interactions has drastic effects on the calculated magnetic properties below 1 K. This is because of the degeneracy breaking of the otherwise degenerate low-lying states. Specific heat is particularly sensitive to rearrangement of low-lying levels and, given that we have *C*(*T*) data down to 0.3 K, this provides a sensitive test.

Full matrix diagonalization of the anisotropic spin Hamiltonian (*i.e.* including the dipolar interactions) is not possible for the full spin system of **1**. Therefore, in order to mimic the effect of the dipolar field on the magnetocalorics of **1** for the full spin system, we have introduced a fictitious effective internal field *B*_eff_ that, by means of its Zeeman splitting, smears out the otherwise sharply peaked density of states. From calculation of *C*(*T*) we find that *B*_eff_ = 0.5 T moves the heat capacity at zero applied field (*B*_0_ = 0) to give good agreement with experiment (Fig. 2c); for an external field of *B*_0_ = 1 T, we find *B*_eff_ = 0.2 T gives good results. For the larger applied fields such a correction is not necessary.

**Fig. 3:**
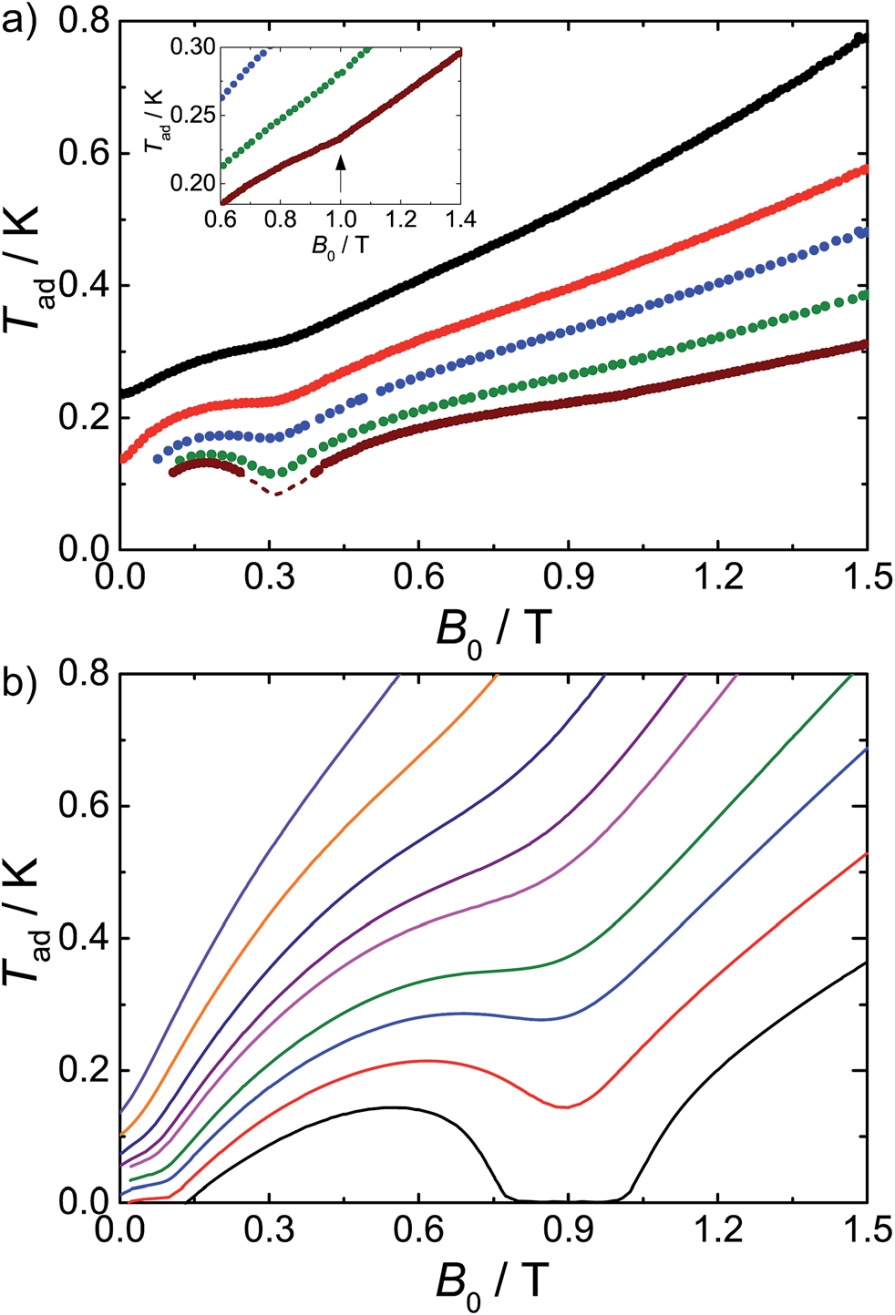
Adiabatic temperature, *T*_ad_, as a function of applied magnetic field, *B*_0_, along isentropic curves: (a) from experiments and (b) calculated using Hamiltonian (1). The dashed line in (a) relates to the uncertainty in the correction applied to the experimental data below 0.1 K (see ESI†). Inset: the arrow highlights the experimental enhancement of *T*_ad_(*B*_0_), which becomes noticeable for *B*_0_ near *ca.* 1 T and *T* < 0.25 K.

We have previously shown that we can observe the effects of spin frustration in the shape of isentropes (paths of constant entropy) in temperature–field plots measured by direct MCE experiments.^7^ Hence, we have performed such measurements for **1** (see ESI† and ref. 6*b* and 7 for the experimental procedure) and also used these to test the influence of dipolar effects on the MCE response. An example of the temperature evolution of **1** on demagnetization under controlled quasi-adiabatic conditions is in Fig. S4†: in this example, demagnetizing from *B*_0_ = 1.5 T results in cooling of the sample from an initial temperature *T*_0_ = 0.6 K to a final temperature *T*_ad_ = 0.13 K, which is significantly lower than the temperatures achieved in our earlier experiments with the planar {Gd_7_} cage.^7^Fig. 3a shows data for different *B*_0_ and *T*_0_, in the form of isentropes (see Fig. S5† for further data).

Unlike a simple paramagnet, where the isentropes would be linear, we find minima in the *T*–*B* plots at *ca.* 0.3 and 1.0 T (the latter is shallow but real). These minima mean there are regions of enhanced and retarded cooling (even warming) on demagnetization. These minima are due to the frustration, and they are most pronounced at the lowest temperatures where quantum effects are dominant, smearing out on warming as many states become Boltzmann populated and the system behaves more classically. Insight into their origin comes from a Zeeman diagram and zero-Kelvin magnetization plot calculated from the Heisenberg Hamiltonian (1) and the isotropic exchange values above (Fig. S6†). Above 1.1 T, there is a unique singly degenerate ground state (nil entropy), *i.e. M* is saturated at 49 *μ*_B_. Below this field there is a high degeneracy of states (high entropy) and the sample cools on demagnetising into this regime. Between *ca.* 0.6 and 0.3 T there is a low (single) degeneracy of states and *M*(*B*) plateaus at 35 *μ*_B_; hence on demagnetising into this region the material passes from high to low entropy which would lead to heating. Below 0.3 T a high degeneracy of states is again met, as the system undergoes a series of level crossings to lower *M*, and we are back in a cooling regime. The 35 *μ*_B_ plateau in *M*(*B*) is consistent with a meta-stable spin configuration where all the outer Gd(iii) spins are fully aligned with each other but fully opposed to the central Gd(iii) spin (an *S* = 35/2 state).

Calculated isentropes based on this Heisenberg model are in good qualitative agreement with the experimental curves (Fig. 3), correctly finding two anomalies in the *T*–*B* curves, but: (i) the theoretical isentropes give the anomalies at *ca. B*_0_ = 0.1 T and 0.9 T, *i.e.* there is a significant field shift particularly for the lower field feature, and (ii) the features, particularly at 0.9 T, are much more pronounced than in the experimental data. Both points can be explained by the neglect of the dipolar interactions in the calculations. In terms of the shift in the lower field minimum, the effect of the dipolar field will be greatest for the lowest applied magnetic fields (and lowest temperatures). This is also apparent from our modelling of the experimental *C*(*T*) data (see above). We also observed such a discrepancy for the centred-hexagonal {Gd_7_} system.^7^ Ultimately, any source of magnetic ordering, including dipolar fields, can shift such features in field. This also limits the lowest attainable temperature upon demagnetization.

The broadening of the isentrope minimum at 0.9 T implies a less regular spin structure than arises from the Heisenberg model, and this cannot be modelled by an additional static field to mimic the dipolar field. Hence, in order to examine this effect of the dipolar interactions we have calculated the *T*–*B* isentropes for the fictitious cluster of *s* = 3/2 spins, enabling exact calculation (see above), with and without dipolar interactions (Fig. S3, ESI†). Inclusion of the dipolar interaction leads to a pronounced smearing of the minimum. Although these results cannot be compared quantitatively to **1** (the different spin leads to different numbers and densities of states, hence to *T*–*B* minima at different fields), the shapes of the curve are remarkably similar to the experimental data for **1**.

## Conclusions

Summarizing, we have found a second example where signatures of geometric spin frustration have been observed in adiabatic demagnetization experiments of a molecular nanomagnet. They give rise to minima in temperature-applied magnetic field curves, in turn giving regions of enhanced cooling at critical applied magnetic fields. In contrast to the previous example,^7^ the isotropic exchange interactions are comparable to the intra-molecular dipolar interactions and the latter cannot be neglected. Their effect is to smear out the frustration signatures and to dampen the enhanced cooling rates. Moreover, although in the present investigation a temperature *T*_ad_ lower than 100 mK could be achieved (Fig. 3), in general dipolar interactions limit the temperatures that can be reached in such experiments. These results highlight the importance of the relative magnitudes of the interaction parameters in molecular clusters in terms of their use for MCE. The antiferromagnetic interactions are necessary for spin frustration which gives rise to large entropy changes, but if the interactions are too large then the full magnetic entropy will not be available on (de)magnetization. If, on the other hand, the interactions are too weak, then the MCE will be limited by dipolar interactions. Most studies of the MCE in molecular systems have simply relied on indirect determination of MCE parameters, and are blind to these effects.

## Supplementary Material

Supplementary information

Crystal structure data
