# Supplementary material for: Observation of the influence of dipolar and spin frustration effects on the magnetocaloric properties of a trigonal prismatic {Gd7} molecular nanomagnet
Source: Chem Sci. 2016 Apr 25;7(8):4891–5. doi: 10.1039/c6sc01415a (PMC6016719; doi:10.1039/c6sc01415a)
Supplement: Supplementary file 1 [file SC-007-C6SC01415A-s001.pdf]

Electronic Supplementary Material (ESI) for Chemical Science.
This journal is © The Royal Society of Chemistry 2016

Supporting Information

Observation of the Influence of Dipolar and Spin Frustration Effects on Magnetocaloric Properties of a Trigonal Prismatic {Gd_7} Molecular Nanomagnet

Eufemio Moreno Pineda,[a] Giulia Lorusso,[b] Karzan H. Zangana,[a,c] Elias Palacios,[b] Jürgen Schnack,[d] Marco Evangelisti,[b] Richard E. P. Winpenny[a*] and Eric J. L. McInnes[a*]

[a] *School of Chemistry and Photon Science Institute, The University of Manchester, Oxford Road, Manchester M13 9PL, UK*

[b] *Instituto de Ciencia de Materiales de Aragón (ICMA), CSIC – Universidad de Zaragoza, Departamento de Física de la Materia Condensada, 50009 Zaragoza, Spain*

[c] *Department of Chemistry, College of Education, Salahaddin University-Erbil, Kurdistan Region-Iraq*

[d] *Faculty of Physics, University of Bielefeld, Universitätsstr. 25, D-33615 Bielefeld, Germany*

Experimental Details

All reagents and metal salts were used as obtained from Aldrich. Analytical data was obtained by the microanalytical service of the University of Manchester.

Preparation of compound 1

Compound **1** was synthesized by refluxing $[Gd_2(O_2C^tBu)_6(HO_2C^tBu)_6]$ (0.469 g, 3 mmol) and diisopropylamine ($^iPr_2NH$) (0.3 mL, 2.0 mmol) in acetonitrile (20 mL) for 3 hrs. The solution was filtered and then allowed to stand undisturbed at ~5 °C for about a week. Hexagonal colorless crystals of **1** suitable for X-ray diffraction were collected: several crystals were measured (giving identical unit cells) to ensure homogeneity of the bulk material. Yield 192 mg (67 %) for **1**, based on $[Gd_2(O_2C^tBu)_6(HO_2C^tBu)_6]$. For EA, the sample was dried at 80 °C for ca. 12 h (resulting in the loss of lattice MeCN). EA for **1** ($Gd_7N_6O_{45}C_{102}H_{204}$), found (calc); C 36.68 (36.73); H 6.15 (6.16); N 2.43 (2.52); Gd 33.04 (33.0). **1** IR (cm^{-1}): 2962.3, 2930.9, 2908.9, 2868.3, 1720.8, 1634.8, 1592.7, 1536.7, 1516.6, 1482.6, 1426.6, 1378.5, 1362.5, 1228.4, 902.0, 791.9. Powdered and crystalline samples gave identical IR spectra (Figure S7).

Crystallography

The data of **1** was collected on Agilent SuperNova CCD diffractometer with MoK_α radiation (λ = 0.71073 Å). The structures were solved by direct methods and refined on *F2* using SHELXTL. CCDC 1458052 contains the supplementary crystallographic data for this paper. These data can be obtained free of charge via www.ccdc.cam.ac.uk/conts/retrieving.html (or from Cambridge Crystallographic Data Centre, 12 Union Road, Cambridge CB21EZ, UK; fax: (+44)1223-336-033; or deposit@ccdc.cam.ac.uk).

Crystal data for **1a** [$C_{106}H_{210}Gd_7O_{45}N_8$]: Mr = 3417.56, hexagonal, $P\bar{6}2c$, T = 150.0(2) K, a = 16.9152(2), c = 31.3646(6) Å, V = 7771.9(3) $Å^3$, Z = 2, ρ = 1.460 g cm^{-3}, total data = 43084, independent reflections 5409(R_{int} = 0.0339), μ = 3.013 mm^{-1}, 305 parameters, R_1 = 0.0310 for $I \geq 2\sigma(I)$ and wR_2 = 0.0637.

Table S1. Continuous shape measures (CShM) for compound **1**.

	**Gd(1)**		**Gd(2)***		**Gd(2)***
**EP-9**	33.545	**OP-8**	35.858	**OP-8**	28.495
**OPY-9**	24.265	**HPY-8**	21.530	**HPY-8**	20.311
**HBPY-9**	21.221	**HBPY-8**	16.427	**HBPY-8**	13.595
**JTC-9**	14.361	**CU-8**	12.942	**CU-8**	11.904
**JCCU-9**	11.215	**SAPR-8**	4.297	**SAPR-8**	3.844
**CCU-9**	10.472	**TDD-8**	2.950	**TDD-8**	3.077
**JCSAPR-9**	1.969	**JGBF-8**	12.364	**JGBF-8**	12.609
**CSAPR-9**	1.305	**JETBPY-8**	26.753	**JETBPY-8**	26.702
**JTCTPR-9**	0.910	**JBTPR-8**	4.751	**JBTPR-8**	3.881
**TCTPR-9**	0.917	**BTPR-8**	4.535	**BTPR-8**	3.079
**JTDIC-9**	13.910	**JSD-8**	5.094	**JSD-8**	4.444
**HH-9**	12.571	**TT-8**	13.356	**TT-8**	12.592
**MFF-9**	2.052	**ETBPY-8**	21.878	**ETBPY-8**	22.549

* one carboxylate is disordered over two positions, related by the σ_h plane, giving rise to two possible geometries at Gd(2): the CShM are given for both possibilities.

EP-9 =(D_{9h}) Enneagon
OPY-9 = (C_{8v}) Octagonal pyramid
HBPY-9 = (D_{7h}) Heptagonal bipyramid
JTC-9 = (C_{3v}) Johnson triangular cupola J3
JCCU-9 = (C_{4v}) Capped cube J8
CCU-9 = (C_{4v}) Spherical-relaxed capped cube
JCSAPR-9 = (C_{4v}) Capped square antiprism J10
CSAPR-9 = (C_{4v}) Spherical capped square antiprism
JTCTPR-9 = (D_{3h}) Tricapped trigonal prism J51
TCTPR-9 = (D_{3h}) Spherical tricapped trigonal prism
JTDIC-9 = (C_{3v}) Tridiminished icosahedron J63
HH-9 = (C_{2v}) Hula-hoop
MFF-9 = (Cs) Muffin
OP-8 = (D_{8h}) Octagon
HPY-8 = (C_{7v}) Heptagonal pyramid
HBPY-8 = (D_{6h}) Hexagonal bipyramid
CU-8 = (Oh) Cube
SAPR-8 = (D_{4d}) Square antiprism
TDD-8 = (D_{2d}) Triangular dodecahedron
JGBF-8 = (D_{2d}) Johnson gyrobifastigium J26
JETBPY-8 = (D_{3h}) Johnson elongated triangular bipyramid J14
JBTPR-8 = (C_{2v}) Biaugmented trigonal prism J50
BTPR-8 = (C_{2v}) Biaugmented trigonal prism
JSD-8 = (D_{2d}) Snub diphenoid J84
TT-8 = (Td) Triakis tetrahedron
ETBPY-8 = (D_{3h}) Elongated trigonal bipyramid

Physical characterization

Susceptibility and Magnetization. The magnetic susceptibility of compound **1** was measurement in the temperature range 1.8 K – 300K employing a powdered sample constrained in eicosane. The measurement was conducted, using a Quantum Design MPMS-XL7 SQUID magnetometer equipped with a 7 T magnet. Magnetization was also collected at 2 and 4 K between 0 and 7 T. The data was corrected for the diamagnetism of the compound (Pascal constants) and for diamagnetic contribution of eicosane and the sample holder. Further magnetization measurements were collected between 0 and 5 T in the temperature range of 1 and 10 K on a Quantum Design SQUID magnetometer MPMS equipped with a magnet operating between 0 and 5 T.

Specific heat. The specific heat (C) measurements were carried out for temperatures down to 0.3 K by using a Quantum Design PPMS, equipped with a ^{3}He cryostat. The experiments were performed on a thin pressed pellet (ca. 1 mg) of a polycrystalline sample, thermalized by *ca.* 0.2 mg of Apiezon N grease, whose contribution was subtracted by using a phenomenonological expression.

Direct magnetization-demagnetization measurements

Direct magnetization-demagnetization measurements of a pressed pellet sample were performed in a commercial ^{3}He setup. The sample-holder consisted of a sapphire plate, with a resistance thermometer (Cernox CX-1010). Wires provided electrical connection, mechanical support and thermal contact to a controlled thermal bath at constant temperature T_0. Each measurement started with the sample at zero applied magnetic field B_0 = 0 and T_0, and comprised the following four steps: (a) gradual application of a magnetic field, up to a maximum B_{max}; (b) relaxation until the sample reached the thermal equilibrium with the bath; (c) gradual demagnetization down to B_0 = 0; (d) relaxation at zero field until the sample reached thermal equilibrium at T_0. During the whole procedure, the as-measured temperature T and applied magnetic field B_0 were recorded at time (t) intervals approximately every second throughout.

In order to cope with the unavoidable lack of adiabatic conditions, we related the as-measured T to the adiabatic temperature T_{ad}, by evaluating the experimental entropy gains (losses) of the sample due to heat dissipated from (to) the thermal bath. Note that the entropy change of the sample in a time interval, $t - t_0$, is

$$\Delta S = \int_{t_0}^{t} \frac{\kappa(T_0 - T)}{T} dT, \tag{1}$$

where κ is the thermal conductance of the wires, which was previously measured as a function of the temperature, using a free-oxygen copper block as the sample. Since the entropy is related to the specific heat by $S = \int(C/T)\mathrm{d}T$ for a constant applied field, deviations of the as-measured temperature from the temperature (T_{ad}) of the corresponding ideal adiabatic procedure yield

$$\Delta S = \int_{T_{ad}}^{T} \frac{C}{T} dT. \tag{2}$$

Therefore, the equivalency between equations (1) and (2) can be used to straightforwardly obtain T_{ad} at every time, knowing κ and C. See, for example, the so-obtained $T_{\mathrm{ad}}(t)$ for the demagnetization depicted in Figure S4. Here, the adiabatic correction to the as-measured temperature is applied for the time interval corresponding to the applied field change from B_0 = 1.5 T to 0, starting from t_0 = 100 s, for T_0 = 0.58 K.

Finally, Figure S5 shows the as-measured field-dependent T data, in the form of isentropes, as obtained on demagnetization (*e.g.*, 100 < t < 260 s in Figure S4), whilst the corresponding temperatures corrected for an ideal adiabatic process, $T_{\mathrm{ad}}(B_0)$, are depicted in Figure 3 of the main text. We mention that T_{ad} is unreliably determined below *ca*. 0.1 K (dashed line in Figure 3 of the main text). This experimental limitation is mainly caused by the fact that the correction is very sensitive to the values of the wires' conductance and specific heat, which approach zero at these low temperatures – therefore, even a small uncertainty could drastically affect T_{ad}.

Numerical simulation

The isotropic, *i.e.*, Heisenberg spin system as well as the anisotropic spin system have been dealt with by exact matrix diagonalization of the respective Hamiltonians. The magnetic observables are then calculated (Figures S2 and S3) using eigenvalues and eigenvectors of the Hamiltonian. In the case of the anisotropic spin system an angular average over 10 directions has been performed for every magnitude of B_0, since the investigated material is a powder of crystallites.

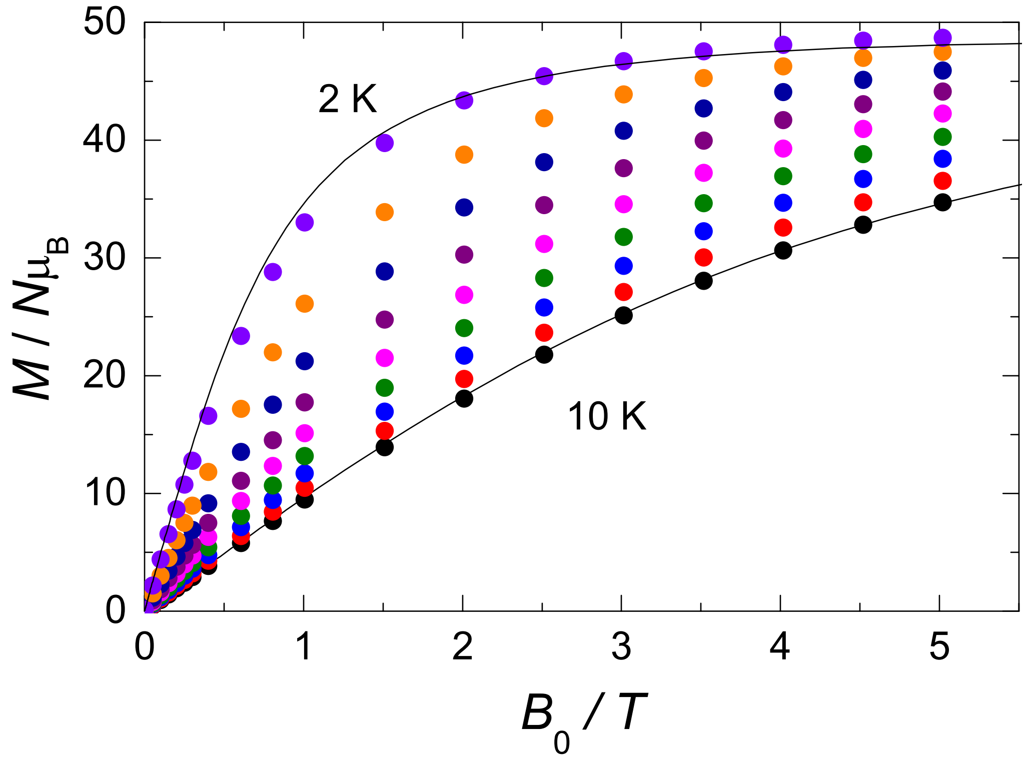

Figure S1. Experimental isothermal molar magnetization data as a function of the applied magnetic field for **1**, collected for temperatures ranging from 2 to 10 K, step 1 K. We apply the Maxwell equation to these data in order to evaluate the magnetic entropy change, which we show in Figure 2 of the main text. Solid lines represent the Brillouin function for seven non-interacting Gd(III) ions.

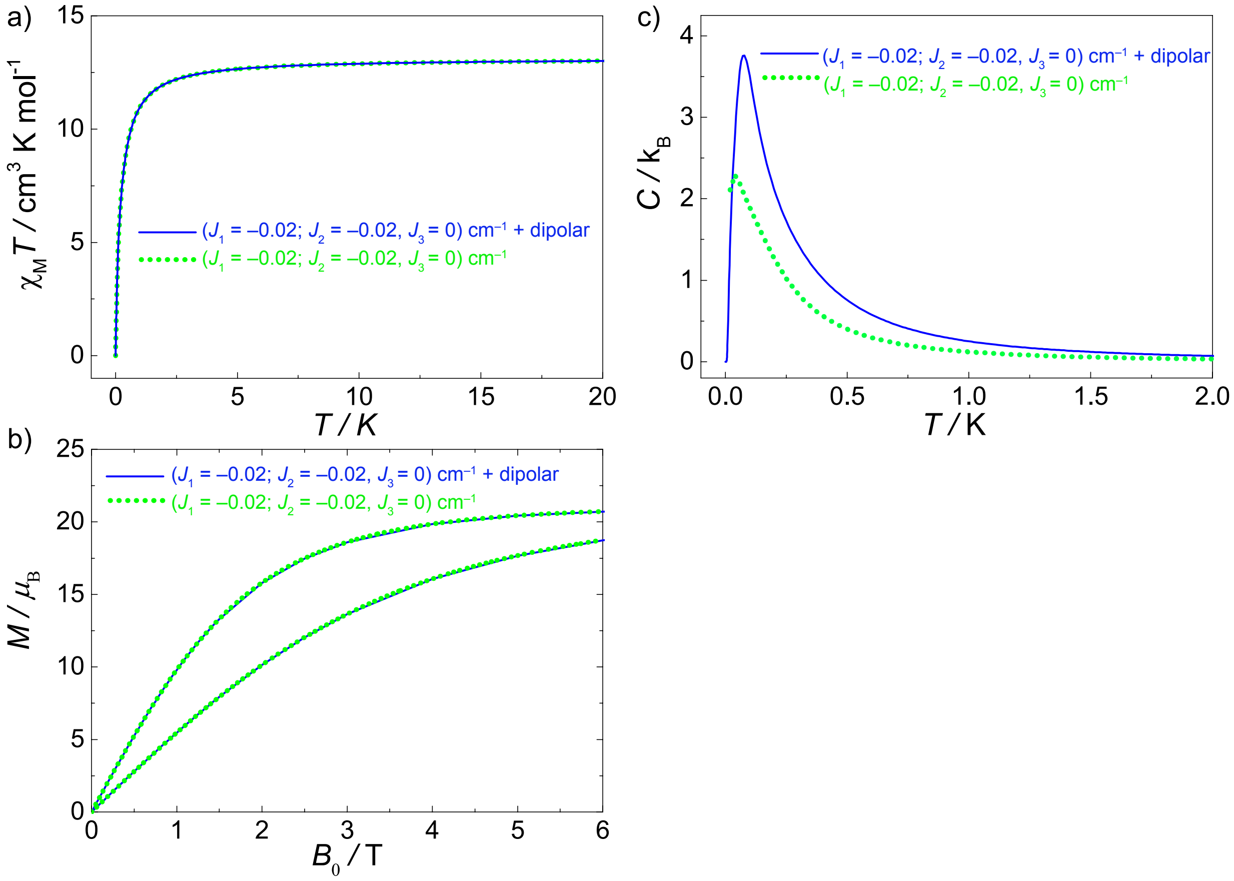

Figure S2. Theoretical magnetic observables for a fictitious cluster, which is isostructural to **1** but has individual spins s = $^3/_2$. Calculations in the Heisenberg model (green dots), calculations for the Heisenberg model and dipolar interactions (blue curves): (a) Molar magnetic susceptibility (χ_M), in the form of $\chi_M T$ as a function of temperature; Magnetization (M) as a function of applied magnetic field (B_0) and temperature (T = 2, 4 K); (c) Specific heat (C) as a function of temperature.

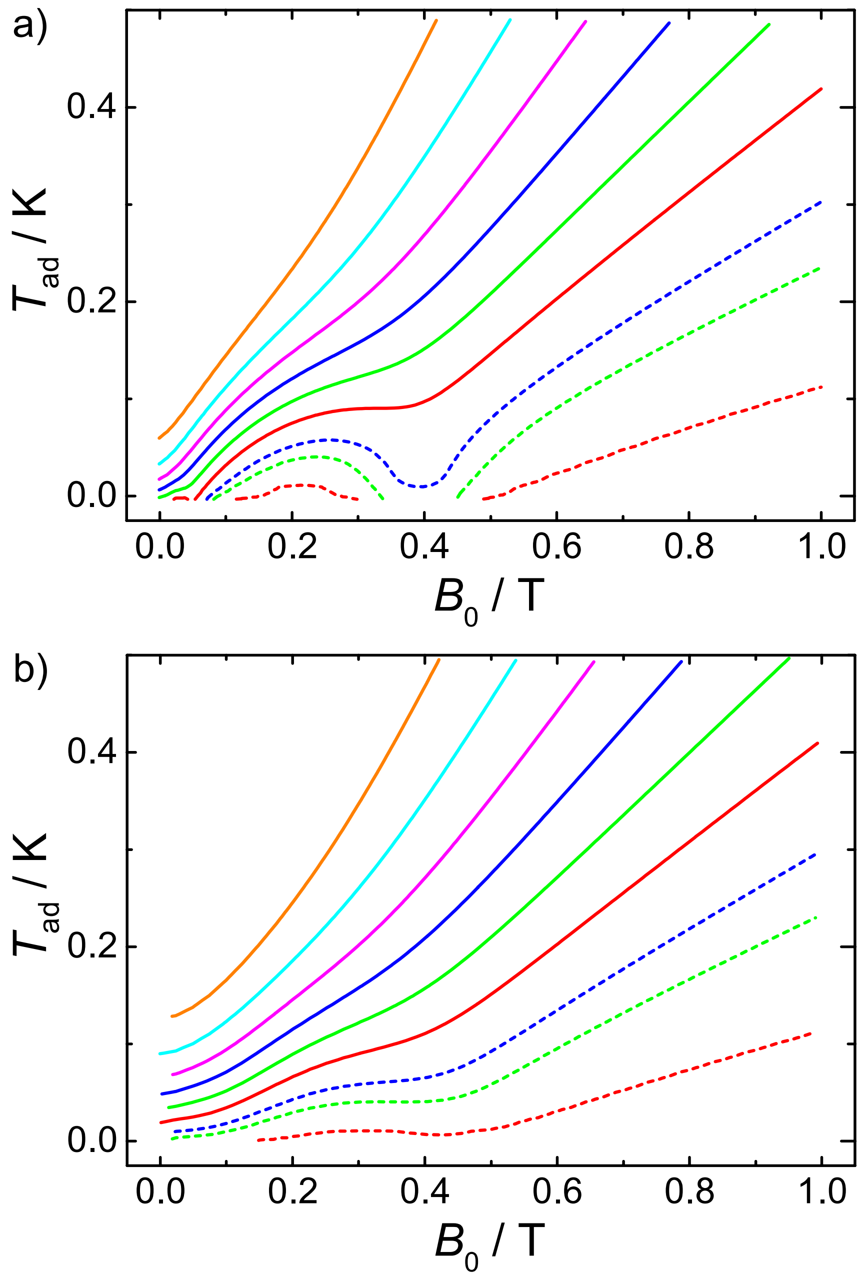

Figure S3. Comparison of isentropes for a fictitious cluster, which is isostructural to **1** but has individual spins $s = {}^3/_2$, using: (a) the Heisenberg model and (b) the dipolar interactions in addition to the Heisenberg model.

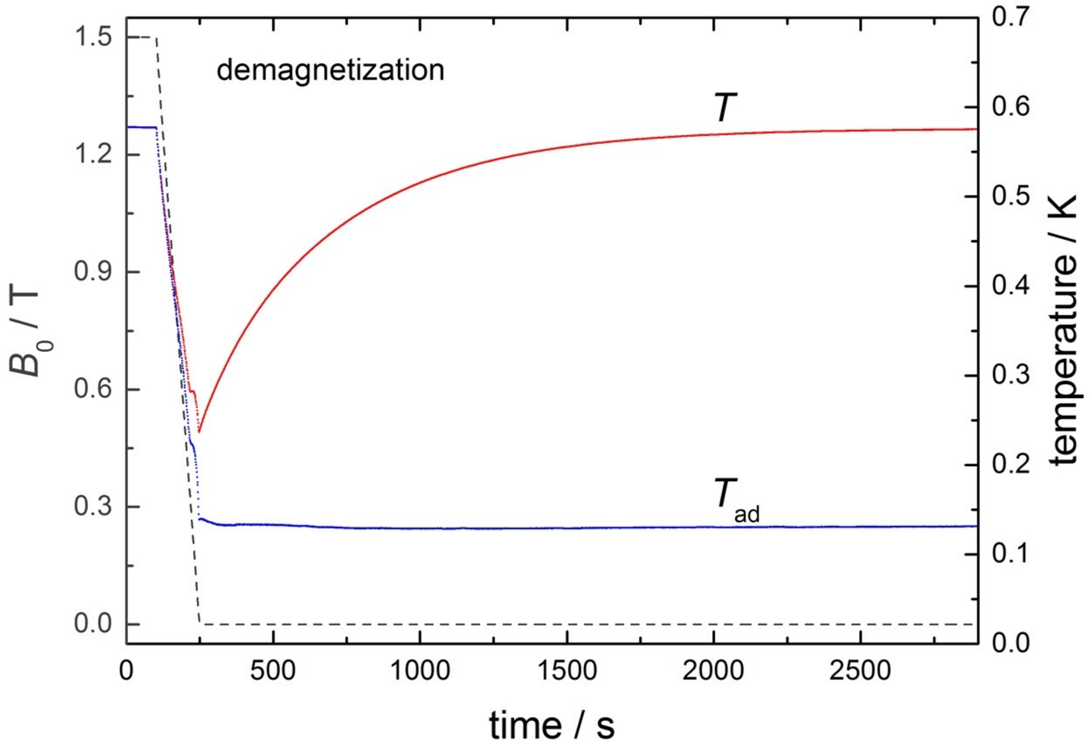

Figure S4. Experimental temperature evolution of **1** on a representative demagnetization, collected for T_0 = 0.58 K and B_{max} = 1.5 T. Shown are the applied magnetic field (B_0; black, dashed line), measured temperature (T; red) and corrected for an ideal adiabatic process (T_{ad}; blue). The experimental temperature is *ca*. 0.23 K at the end of the demagnetization (260 s), then it gradually reverts back to the initial value (reached after *ca*. 3000 s) because of heat dissipation (mainly via the wires holding the sample platform). The corrected adiabatic temperature reaches *ca*. 0.13 K at the end of the demagnetization and holds this value constant, as expected for constant applied field (B_0 = 0).

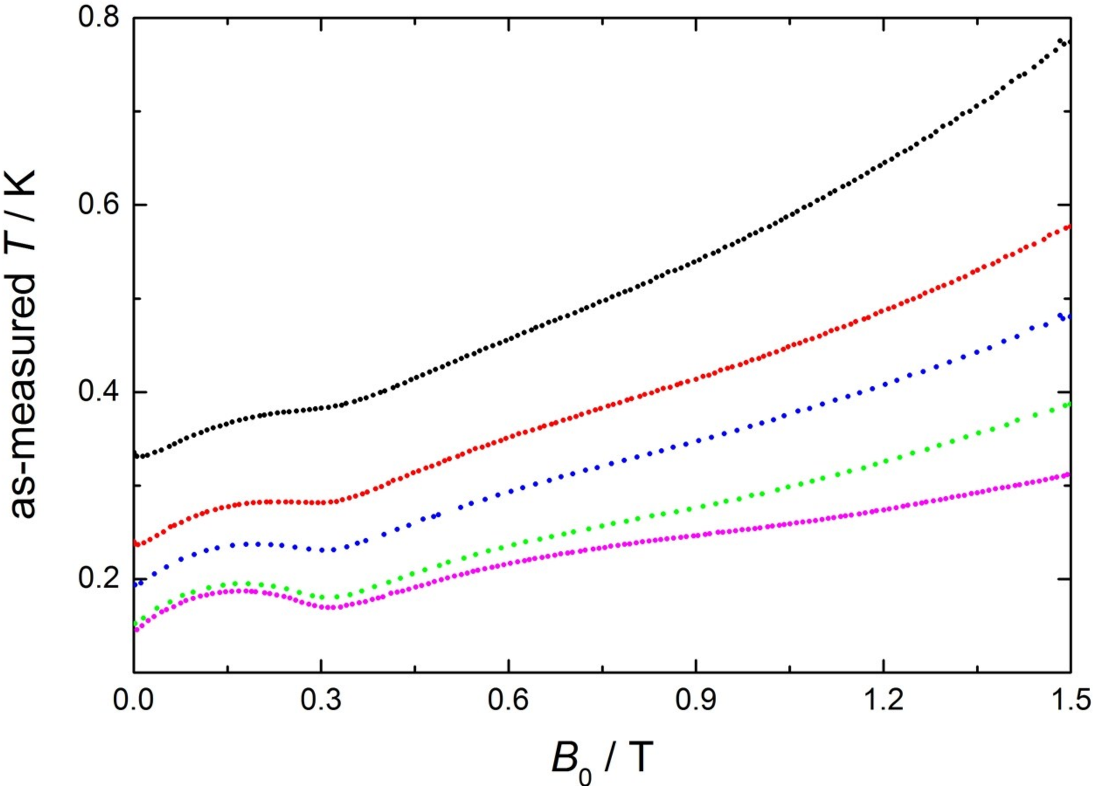

Figure S5. Experimental field evolution of the as-measured temperature for **1**, as collected for B_{max} = 1.5 T and different values of the starting temperature, T_0, for the demagnetization. Note that the red curve (T_0 = 0.58 K) represents the same set of data shown in Figure S4.

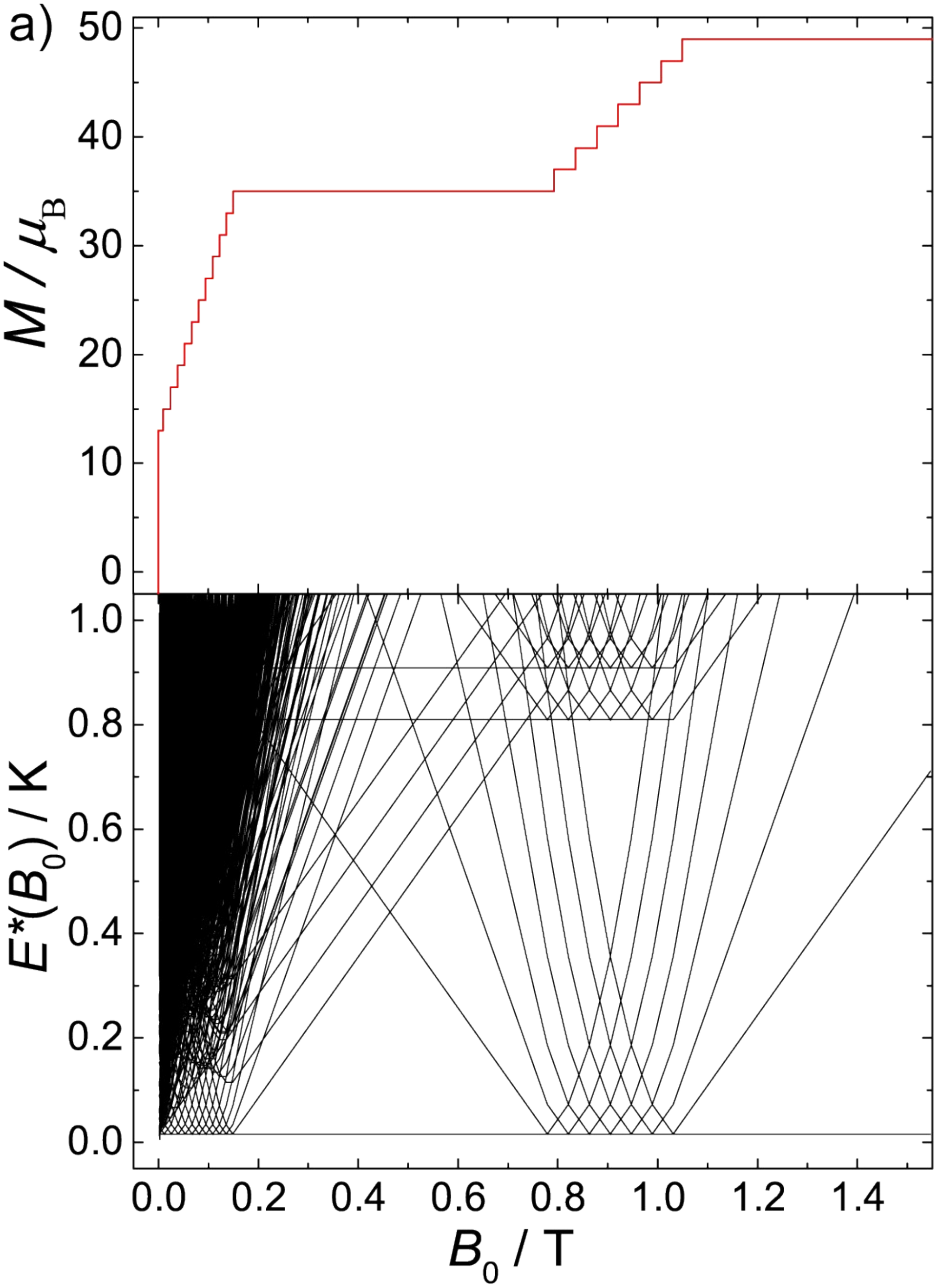

Figure S6. (a) Theoretical magnetization curve of **1** at T = 0 calculated for the Heisenberg Hamiltonian (1) of the main text. The step structure emerges from ground state level crossings, which are depicted in (b). (b) Zeeman diagram for **1**, as calculated using the Hamiltonian (1) of the main text. Shown are excitation energies E^* = (E_i – E_o) *vs.* B_0, where E_i and E_o are the energies of the ith and ground Zeeman states, respectively, at any given B_0. The relatively higher density of low-lying states in proximity of *ca*. B_0 = 0.1 T and 0.9 T gives origin to enhanced cooling rates at these field values, as shown by the calculated isentropes in Figure 3 of the main text.

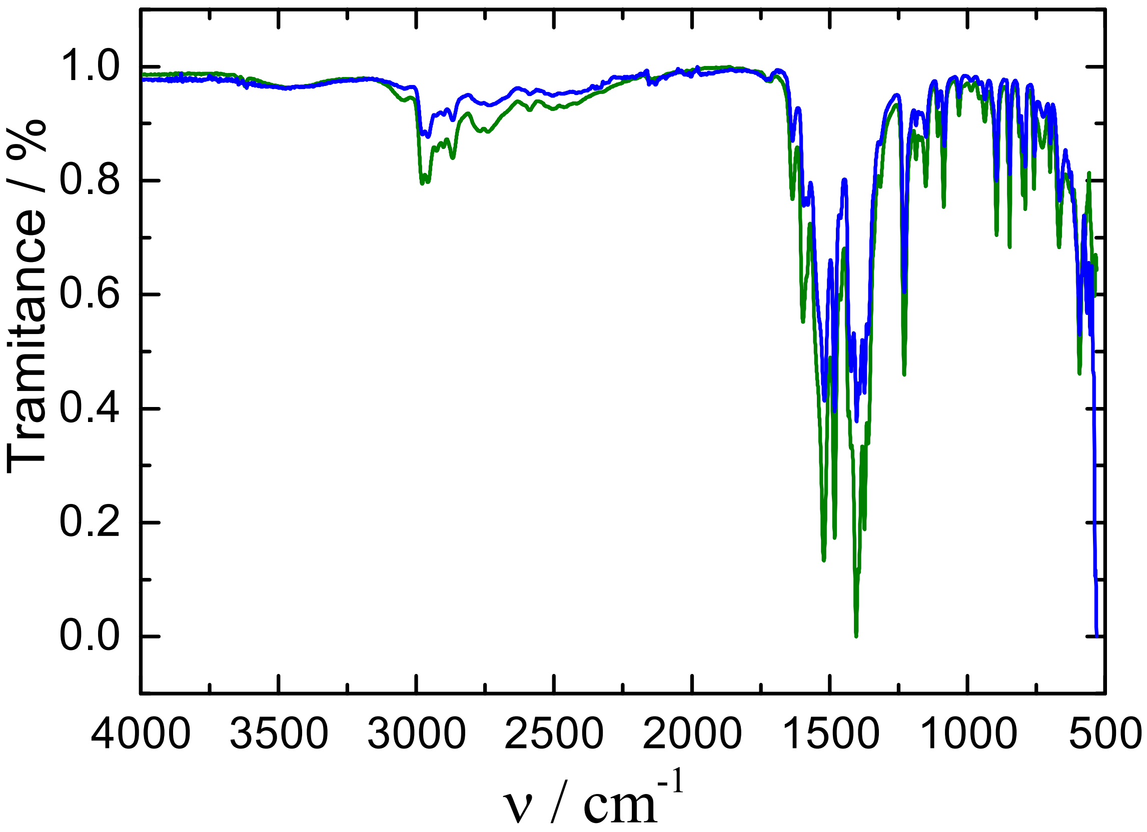

Figure S7. Solid state infrared spectrum of **1** of powder (green) and crystals (blue).
